# The ASCO Study of Collaborative Practice Arrangements: A Nurse Practitioner’s Point of View

**Published:** 2012-01-01

**Authors:** Hollie Devine

**Affiliations:** From The Ohio State University Medical Center, Columbus, Ohio

As a veteran oncology nurse practitioner, the findings presented by Towle et al. (2011) in their article, "Results of the ASCO Study of Collaborative Practice Arrangements," recently published in the *Journal of Oncology Practice*, were not astonishing to me. The purpose of their study was to address the workforce shortage of oncologists by exploring collaborative oncology practice models that include nonphysician practitioners (NPPs)—physician assistants and nurse practitioners. The study design consisted of two steps: (1) a national survey of NPP integration into oncology practices and identification of collaborative practice models and services, and (2) a more detailed data and satisfaction survey of selected practices.

## Study Overview

Beginning in 2009, a total of 226 community oncology practices representing 43 states participated in the study; academic oncology practices were excluded. Fifty-eight percent of the medical oncology practices confirmed integration of NPPs in collaborative practice agreements. As a result of those responses, 27 practices from 24 geographically distinct states were selected for further inquiry. Although many of my fellow NPPs have provided data to their respective physicians and leadership championing the rationale for hiring and on-boarding additional NPPs in their respective practices, Towle et al. have further provided medical oncologists with evidence-based data on the positive impact NPPs have in oncology practices.

The results of this study provided the medical oncology community with compelling data regarding investing in NPPs. First, this study demonstrated that oncology patients know who and what NPPs are, and that our contributions to their care yield high satisfaction scores. As a nurse practitioner, my practice is grounded in the principles of nursing. Although I can assess, diagnose, and treat, I am also an advocate and a counselor for the patients as well as their care provider. While I have not personally experienced cancer, I have walked alongside thousands of patients with hematologic malignancies. Whether it is a gesture, a smile, a gentle touch, or just being present, oncology nurse practitioners continue to demonstrate compassion and excellence in patient care.

## Effective Partnerships

Both physicians and NPPs surveyed in this study were satisfied with the level of professionalism in their respective collaborative practices. Personally, I owe my humble beginnings to my first collaborative practice physician. I was transitioning from an inpatient bedside nurse role to being the first nurse practitioner in an oncology practice. My collaborating physician identified the knowledge, skills, and other attributes that I, as an oncology nurse, could contribute to his practice and he agreed to mentor me in the domain of advanced practice.

In that role, I was able to demonstrate that I could assume some of the physician’s responsibilities. As a team member, I could assist in patient care in the ambulatory and hospital settings, perform procedures, as well as assist with toxicity grading for patients on clinical trials. Hence I was able to think critically and expand my skill set as well as free up my collaborative physician’s time to focus on new patient consultations; complex patient cases; and academic, research, and administrative responsibilities. In time, I had my own clinic and was financially contributing to the growth of the practice. Currently, with NPPs billing incident-to, reimbursement and resource utilization further impels the collaborative practice model. For example, NPPs can bill for their services, such as routine ambulatory and hospital patient visits, symptom management, patient education and counseling, procedures, and on-call activities.

Currently, health-care resources are fixed. However, Towle et al. noted that productivity was enhanced by 19% when NPPs worked with multiple physicians rather than exclusively with one physician. Working as a team with my collaborating practice physician, we are able to "divide and conquer." Each of our roles within the health-care team provides a unique perspective that makes our patients feel secure and cared for. With the deficit of projected oncologists, this study provides insight into the productivity contributions NPPs can make to an oncology practice. Although NPPs from the academic environment were not included in this study, restrictions on medical residents’ caseloads and working hours will continue. In light of these restrictions, the use of NPPs is projected to increase further.

## Training and Support

A topic that warrants more discussion is the training model identified for oncology NPPs. According to the survey, a majority of the ASCO members used informal/on-the-job training programs for new NPPs, followed by practices only hiring NPPs with previous hematology/oncology experience. I was an experienced oncology nurse transitioning into an advanced practice role, and I did receive on-the-job training from a physician. However, this was due to the fact that at that time, nurse practitioners were novel to oncology practices.

Oncology is a specialty within nursing and advanced practice, as evidenced by the membership of the Oncology Nursing Society (ONS). When I began my advanced practice career in 1997 as well as today, the ONS has been my foundation and a pivotal resource. The ONS has developed and implemented conferences, continuing education sessions, skills workshops, and special interest groups, with a focus on advanced practice. Leadership, mentoring, evidence-based practice, and research are other areas that the ONS supports in advanced practice. These are noteworthy resources for the advanced practice nurse new to either oncology or advanced practice.

Oncology training programs through colleges of nursing as well as National Cancer Institute (NCI)-designated cancer centers are other avenues for advanced practice clinicians to gain additional training, mentoring, and expertise in oncology. Finally, many NCI-designated cancer centers, practices, and even state regulations identify the advanced oncology nurse certification through the ONS.

## Conclusion

Oncology nurse practitioners must be cognizant of their discipline’s scope of practice as well as rules and regulations, since they vary state by state. Regardless of practice location, providing excellence in oncology patient care is what we do, and what our fellow clinical nurse specialists, nurses, physician assistants, patients, and their care providers have observed us doing. It is timely and appreciated that our oncology physician counterparts and their professional organizations acknowledge and consider our roles and contributions, despite the fact that it is what we have been doing for decades.

## References

[1] Towle Elaine L, Barr Thomas R, Hanley Amy, Kosty Michael, Williams Stephanie, Goldstein Michael A (2011). Results of the ASCO Study of Collaborative Practice Arrangements.. *Journal of oncology practice / American Society of Clinical Oncology*.

